# RNAct: Protein–RNA interaction predictions for model organisms with supporting experimental data

**DOI:** 10.1093/nar/gky967

**Published:** 2018-11-16

**Authors:** Benjamin Lang, Alexandros Armaos, Gian G Tartaglia

**Affiliations:** 1 Centre for Genomic Regulation (CRG), The Barcelona Institute of Science and Technology, Barcelona 08003, Spain; 2 Institució Catalana de Recerca i Estudis Avançats (ICREA), 23 Passeig Lluís Companys, Barcelona 08010, Spain; 3 Universitat Pompeu Fabra (UPF), Department of Experimental and Health Sciences, Barcelona 08003, Spain; 4 Department of Biology ‘Charles Darwin’, Sapienza University of Rome, P.le A. Moro 5, Rome 00185, Italy

## Abstract

Protein–RNA interactions are implicated in a number of physiological roles as well as diseases, with molecular mechanisms ranging from defects in RNA splicing, localization and translation to the formation of aggregates. Currently, ∼1400 human proteins have experimental evidence of RNA-binding activity. However, only ∼250 of these proteins currently have experimental data on their target RNAs from various sequencing-based methods such as eCLIP. To bridge this gap, we used an established, computationally expensive protein–RNA interaction prediction method, *cat*RAPID, to populate a large database, RNAct. RNAct allows easy lookup of known and predicted interactions and enables global views of the human, mouse and yeast protein–RNA interactomes, expanding them in a genome-wide manner far beyond experimental data (http://rnact.crg.eu).

## INTRODUCTION

RNA-binding proteins (RBPs) are key in RNA splicing, processing, export, localization and regulation of translation and are implicated in a number of pathologies in humans. Examples include heterogeneous and life-threatening genetic disorders, such as amyotrophic lateral sclerosis (1), spinocerebellar ataxia and retinitis pigmentosa, among others (2,3). Human proteins encoded by 1393 genes currently have experimental evidence of RNA-binding activity (4–6). These proteins contain one or more RNA-binding regions, either in the form of canonical globular domains or of more recently discovered, intrinsically disordered RNA interaction regions (7,8). Additionally, protein–protein interaction interfaces and enzymatic active sites are sometimes employed for RNA binding (4,9). Protein–RNA interactions form an intricate network, and RNAs play structural roles in many types of phase-separated biological condensates, such as stress granules (10).

However, the number of RBPs for which the identity of their interaction partners is known is much lower. Two hundred fifty *Homo sapiens* RBPs currently have high-throughput experimental data on the identity of their target RNAs (11,12), obtained mostly by various sequencing-based methods such as eCLIP, iCLIP, HITS-CLIP, PAR-CLIP and RIP-seq. Much smaller datasets are available for *Mus musculus* (38 RBPs (12)), *Drosophila melanogaster* (29 RBPs from RIP-seq (13)) and *Saccharomyces cerevisiae* (69 RBPs from RIP-Chip (14)). A comprehensive collection of CLIP data is available in the recently expanded POSTAR database (12), previously called CLIPdb, which also includes motif-based target predictions for a set of human and mouse RBPs (88 and 82, respectively).

To bridge the gap between the 1393 known RBPs and the 250 for which we have experimental knowledge of interaction partners, we used an established, experimentally validated (15,16) protein–RNA interaction prediction method, *cat*RAPID (17–19), to generate proteome- and transcriptome-wide sets of interaction predictions. Our database now covers the *H. sapiens, M. musculus* and *S. cerevisiae* genomes and contains a total of 5.87 billion pairwise interactions. This reflects nearly 120 years of computation time on the Centre for Genomic Regulation's high-performance computing cluster, and for the first time provides all possible protein–RNA interactions in these species.

RNAct makes available our genome-wide protein–RNA interaction predictions and combines them with powerful and intuitive search functionality, including pairwise search for sets of proteins and RNAs. The display is enriched with useful annotation, including transcript support level (TSL) and APPRIS classification for isoforms and RNA subcellular localization from the RNALocate database. Known RBPs as well as interactions confirmed by large-scale experiments from the ENCODE project are clearly highlighted.

## MATERIALS AND METHODS

### Proteomes

Proteomes were obtained from UniProt (20). Sequence files containing all canonical sequences from each organism’s reference proteome were obtained from the UniProt FTP server (these exclude the ‘additional’ isoform transcripts for a given UniProt accession). This resulted in successful interaction predictions for 20 778 canonical human proteins (proteome UP000005640 from UniProt release 2017_10), 22 080 canonical mouse proteins (proteome UP000000589 from UniProt 2018_01, strain C57BL/6J) and 5 963 canonical yeast proteins (proteome UP000002311 from UniProt 2018_06, strain ATCC 204508 / S288c).

### Transcriptomes

Transcriptomes were obtained from GENCODE (for human and mouse) (21) and Ensembl (for yeast) (22). GENCODE ‘basic’ RNAs are a representative subset prioritizing full-length protein-coding transcripts over partial or non-coding transcripts for a given gene. The GENCODE release used for human is Release 27 (genome assembly GRCh38.p10), and both the ‘basic’ (98 608 transcripts with successful interaction predictions) and ‘non-basic’ (100 722 transcripts) subsets were obtained for full coverage of the human GENCODE transcriptome. These sets are kept separate for performance reasons, and the protein view currently does not show non-basic human RNAs (except in the pairwise search). For mouse, GENCODE release M16 (genome assembly GRCm38.p5) was used, retaining only the ‘basic’ subset (76 532 transcripts, ∼58% of the mouse GENCODE transcriptome) due to resource and computation time constraints. For yeast, all coding and non-coding transcripts from the Ensembl 92 release (April 2018) were included (7029 transcripts with successful interaction predictions).

All FASTA sequence files used are available for download in the RNAct Download section. A small number of these sequences were excluded from RNAct due to limitations of the *cat*RAPID algorithm: short or extreme length (proteins ≤50 aa or >14 507 aa, RNAs ≤50 nt or >28 227 nt), or unsuccessful RNA secondary structure prediction using the ViennaRNA package which *cat*RAPID relies on (23).

### Interaction predictions (*cat*RAPID maximum fragment score)

**Figure 1.**
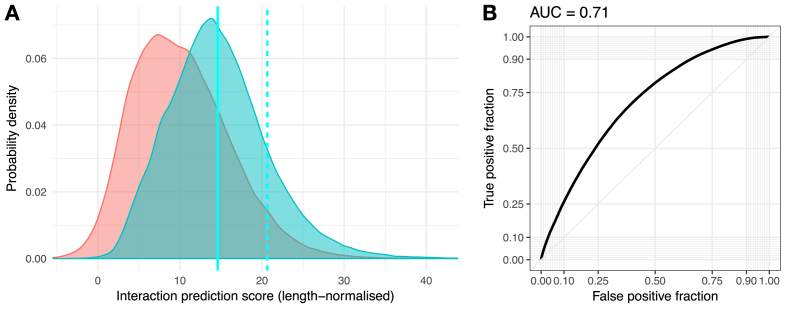
(**A**) Interaction propensity scores for the background (sampled from slightly over 2 billion human protein–RNA pairs; light red) and positive set (212 256 high-confidence protein–RNA interactions revealed by eCLIP; cyan). The *z*-score reported in the results pages is computed on the right-skewed blue distribution, with the solid cyan line indicating the mean and the dashed line indicating a *z*-score of 1 (one standard deviation above the mean). (**B**) The area under the ROC curve of 0.78 (0.72 upon length normalization) indicates the predictive performance of the *cat*RAPID method on recent high-confidence experimental eCLIP data from the ENCODE project.

To compute the interaction propensity scores, we used the *cat*RAPID approach (17) with the fragmentation procedure (18,19) and normalized for sequence lengths (19). For each protein–RNA pair, the fragments with the maximum interaction propensity score are used to assess overall binding ability (Figure 1A). The *cat*RAPID score shows a receiver operating characteristic (ROC) area under the curve (AUC) of 0.78 with high-confidence eCLIP data (212 256 interactions with human GENCODE ‘basic’ RNAs, replicated in at least one cell line studied in ENCODE and in all replicates in each).

When including all eCLIP interactions regardless of replication (723 881 interactions for GENCODE ‘basic’ RNAs), this AUC is still 0.76. Normalizing the prediction score by sequence lengths, similarly to a previous work (19), we found that the predictive performance decreases slightly (to an AUC of 0.71 on the high-confidence interactions, and of 0.70 on all). This indicates a size effect, potentially due to the RNAse digestion step in CLIP protocols. We stress that the method was trained on X-ray and NMR data, and that its performance on the experimental CLIP data reflects its predictive power (Figure 1B). RNAct displays the length-normalized prediction scores, with raw *cat*RAPID scores available for download upon request.

### Experimental interaction data (ENCODE eCLIP)

Experimental interaction data covering 119 human RBPs using eCLIP in the HepG2 and K562 cell lines (170 total experiments) were obtained from the ENCODE Project in narrowPeak format (11,24,25). This represents the largest single dataset of experimental protein–RNA interaction data currently available. Additional experimentally determined interactions covering 69 RBPs in yeast using RIP-Chip were obtained from a compilation by Mittal *et al.* (14).

### Protein and RNA annotation

A very recent census of proteins with experimental evidence of RNA-binding activity in human (1393 known RBP genes), mouse (1914 known RBPs) and yeast (1273 known RBPs) was used to flag proteins as known RBPs in RNAct (4). Additionally, an older census of 1542 RBPs, which used features such as domain composition and known roles of proteins, was used to flag a further 658 human RNAct proteins as known RBPs (3). Overall, 5097 proteins in RNAct are flagged as ‘Known RBPs’, with 2031 of these being human.

In addition to annotated, known RBPs, we obtained predictions of RNA-binding activity from SONAR (26) (1923 predicted human RBPs) and *cat*RAPID signature (27). *cat*RAPID signature was used with a threshold score of 0.735, equivalent to a *z*-normalized value of 1 (one standard deviation above the mean) for the score distribution for known human RBPs from Hentze *et al.* (4), resulting in 1268 predicted human RBPs. Overall, 2779 human proteins in RNAct are flagged as ‘Predicted RBPs’, 1721 of these being novel (not ‘known’).

RNA subcellular localization was obtained from the RNALocate database with very minor curation, removing a handful of ambiguous or non-subcellular terms (28). Basic protein annotation including gene symbols, full protein names and sequence length was obtained from UniProt. RNA annotation including transcript symbols (e.g. ‘TARDBP-201’), length, biotype (e.g. ‘protein coding’, ‘lincRNA’), GENCODE ‘basic’ status and TSL were obtained from GENCODE and Ensembl. Principal (primary) and alternative isoform classifications were obtained from APPRIS (29).

### Technical aspects

RNAct is implemented in PHP on an Apache server using a MariaDB SQL backend, storing ∼450 GB of pre-sorted tables. The interaction predictions were calculated over several months on a shared set of 80 HP BL460c nodes with two Intel Xeon E5-2680 2.70 GHz CPUs and 120 GB of usable DDR3-1600 memory each, using 8 cores per cluster job. These are part of the CRG’s high-performance computing cluster. The open-source Bootstrap library was used to ensure correct display on devices of any screen size, including mobile devices. Several icons were included from Font Awesome and the Noun Project (please see the About section of the website for attributions). RNAct collects no data on its users.

## USING RNAct

### Search functionality

**Figure 2.**
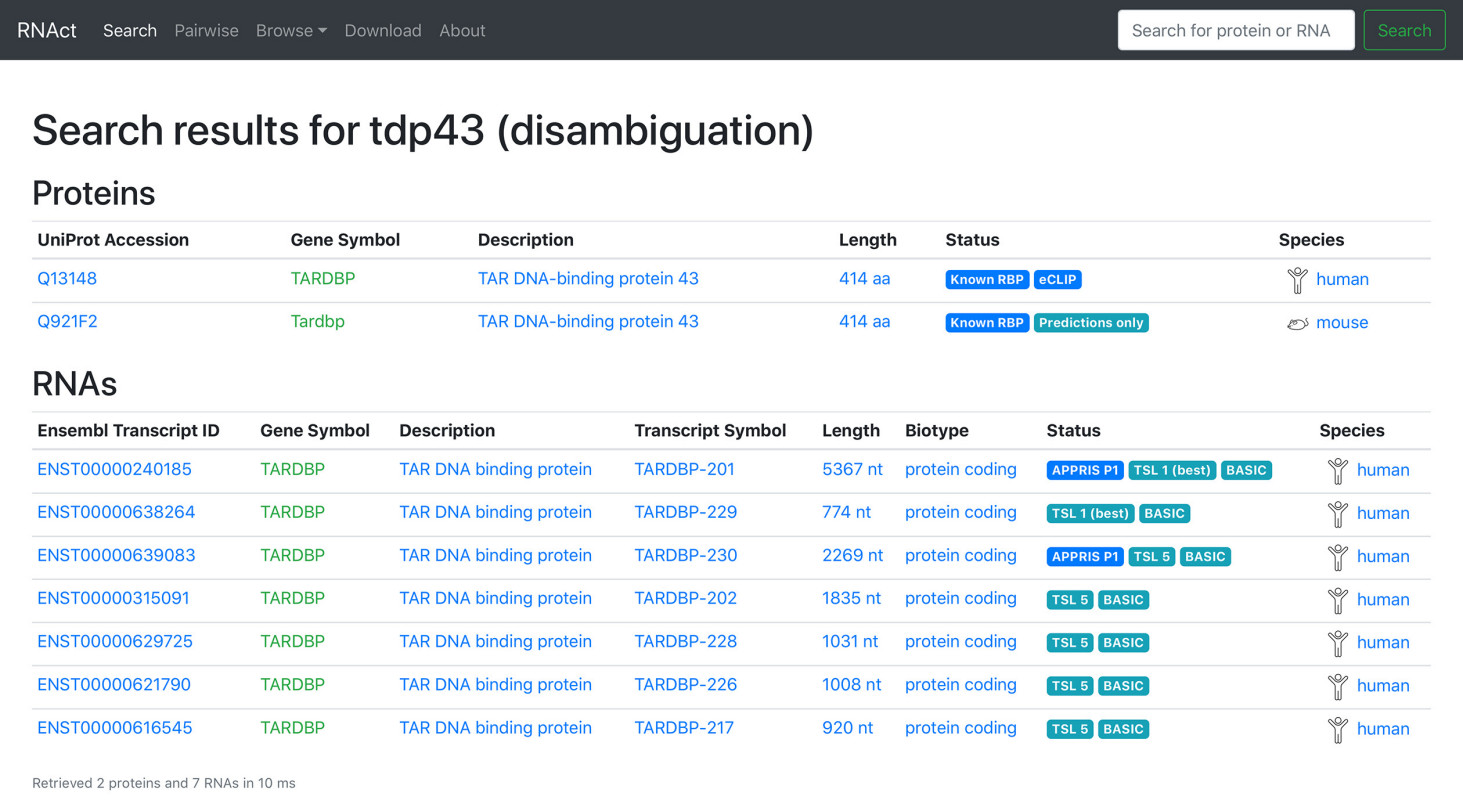
Search results (disambiguation page). This page allows selection of the protein or RNA of interest across the 3 species currently in RNAct.

RNAct is built for extreme ease and speed of real-world use. The landing page (Search) contains a single search box which allows entry of any protein or RNA identifier (e.g. ‘tdp43’ or ‘hotair’). Unless the term is highly ambiguous (e.g. ‘ataxin’), most searches resolve to a single gene symbol, giving a choice of species and protein or RNA on the disambiguation page that follows (Figure 2). Table 1 shows a list of realistic search terms that are resolved successfully by RNAct. This is achieved by ‘guessing’ the identifier type, moving outwards from specific to more ambiguous options, if necessary. There is no built-in limit to the number of search results returned, allowing searches for e.g. ‘RNA binding’, ’vault RNA’ or ‘lysine demethylase’.

**Figure 3.**
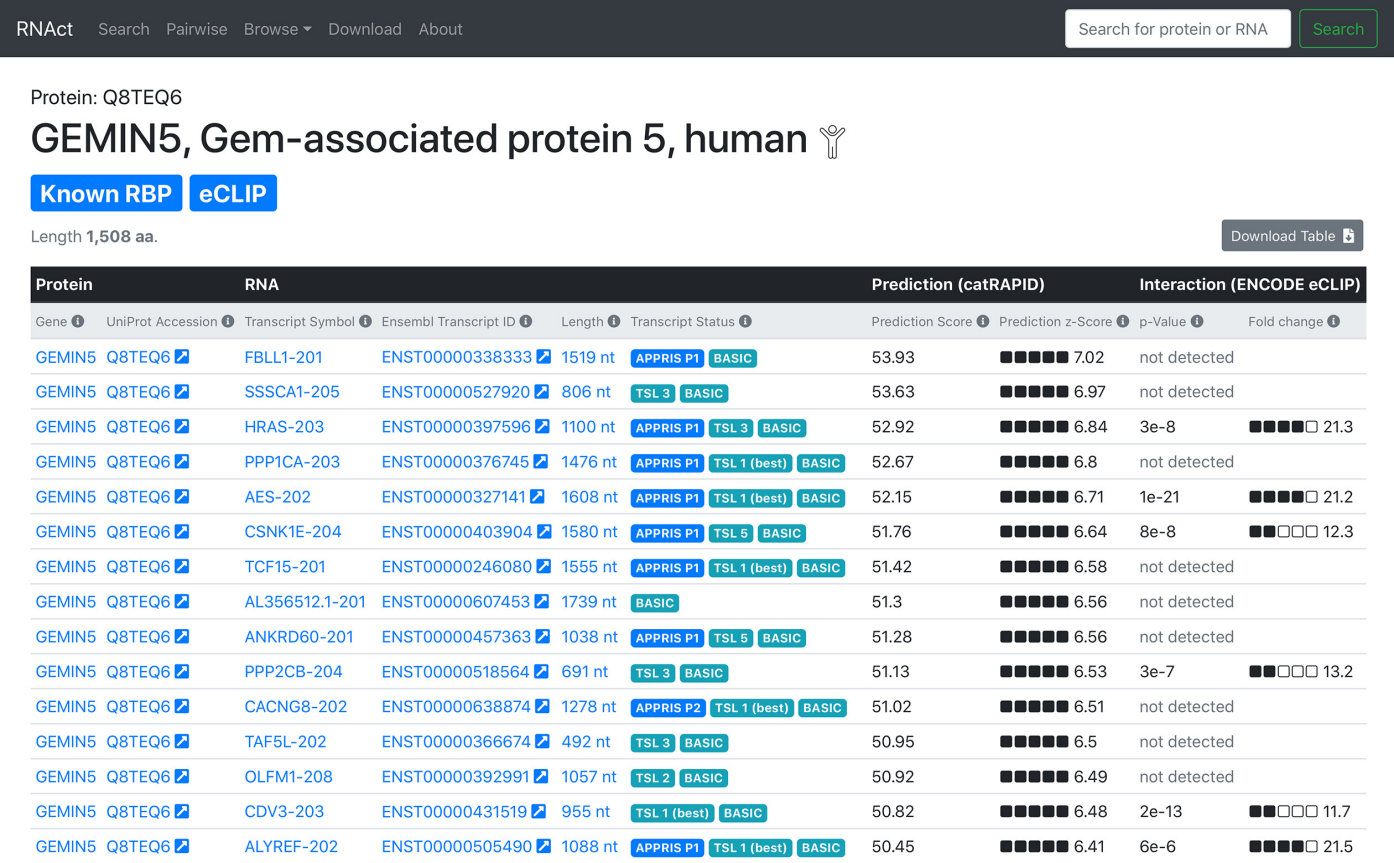
The Protein view. This page shows a list of potential RNA interaction partners prioritized by *cat*RAPID length-normalized prediction score. Alternatively, the page can be sorted by eCLIP experimental results by clicking on the ‘*P*-value’ or ‘fold change’ columns. Useful information on the protein of interest, such as whether it is a known or predicted RBP and whether experimental interaction data (e.g. from eCLIP experiments) exists for it is shown at the top of this view, and transcript annotation and quality information are shown as badges for each RNA. Links out to Ensembl and UniProt are provided. Other links lead to the protein’s or RNA’s view within RNAct.

**Table 1.** Examples of realistic search terms successfully resolved by RNAct

Real-world search term	Retrieved gene symbol(s)	Retrieved description	Retrieved via
‘annexin 11’	ANXA11	Annexin A11	Partial description match
‘ews’	EWSR1	RNA-binding protein EWS	Gene symbol alias
ENSG00000089280	FUS	RNA-binding protein FUS	Ensembl gene identifier
FUS_MOUSE	FUS	RNA-binding protein FUS	UniProt identifier
P35637	FUS	RNA-binding protein FUS	UniProt accession
‘pur α’	PURA	Transcriptional activator protein Pur-α	Partial description match
‘smn’	SMN1	Survival motor neuron protein	Partial symbol match
	SMNDC1	Survival of motor neuron-related-splicing factor 30	
‘tdp43’	TARDBP	TAR DNA-binding protein 43	Gene symbol alias, ignoring punctuation (via TDP-43)

This design minimizes tedious input elements (e.g. a species dropdown box) and instead facilitates discovery and comparison across protein families and species. Matching fields are highlighted in green, which allows intuitive selection of the intended match (e.g. the RNA transcript in question when searching for ‘ENST00000237536’) while leaving room for additional useful choices (e.g. the corresponding protein for transcript ‘ENST00000237536’). The search box is available in the top right of every page and is easily navigated to by pressing the tab key.

### Protein view

Once a protein of interest is selected, the Protein view (Figure 3) shows a list of RNA interaction partners prioritized by prediction score. Alternatively, the view can be sorted by experimental results simply by clicking on the experimental columns. The length, GENCODE ‘basic’ status, APPRIS classification and TSL (22) for each transcript are shown, allowing isoform quality assessment. Links out to Ensembl and UniProt for additional transcript and protein information respectively are provided (with an arrow symbol).

### RNA view

Once an RNA is selected, the RNA view shows a list of predicted protein interaction partners prioritized by prediction score. Alternatively, the view can be sorted by experimental results simply by clicking on the experimental columns. Interactions with experimental evidence are highlighted (14,24), as are known (3,4) and predicted (26,27) RBPs. Links out to Ensembl and UniProt for additional information are provided.

### Advanced pairwise search

A common use case for RNAct is the prediction of interactions within a set of proteins and RNAs, allowing the rapid prioritization of candidates for validation, and the analysis of specific pathways or systems. The Pairwise search feature allows entry of a set of proteins and a set of RNAs, either in multiple lines or separated by commas, and allows any identifier types which the Search function can resolve, including ambiguous queries (e.g. for ‘lysine demethylase’). The only limitation is the total number of pairs queried, which is currently limited to 10 000 (allowing entry of e.g. 100 proteins and 100 RNAs).

### Browse proteins or RNAs

These views list all proteins or RNAs contained in RNAct, i.e. the human, mouse and yeast reference proteomes and transcriptomes. In the Browse Proteins view, proteins are listed in order of availability of experimental interaction data (e.g. from eCLIP), evidence of RNA-binding activity (known or predicted RBPs), species and gene symbol. This allows the easy retrieval of known RBPs, particularly those with experimental interaction data. In the Browse RNAs view, transcripts are sorted by species, gene symbol, GENCODE ‘basic’ status, APPRIS classification, TSL and descending transcript length. This means that the best-supported transcript for a given gene will appear first.

### Download

All RNAct protein–RNA interaction prediction data for human, mouse and yeast are available from the Download page. For human, the predictions are split into two sets for performance reasons: GENCODE ‘basic’ transcripts (covering a representative subset of 98 608 RNAs), and ‘non-basic’ transcripts making up the rest of the transcriptome. Both files can be concatenated for a full view of the human protein–RNA interactome, covering 20 778 proteins and 199 330 RNA transcripts. For mouse, only the GENCODE ‘basic’ transcripts are currently available, while the full annotated transcriptome is available for yeast. The RNAct predictions are licenced under a Creative Commons Attribution-NonCommercial-ShareAlike 4.0 International Licence (CC BY-NC-SA 4.0). A complete set of supporting tables containing protein and RNA annotations, identifier mappings used internally for searching, and the experimental data used (e.g. eCLIP) is available on the Download page as well. We intend to complete and add predictions for additional species such as *C. elegans* and *D. melanogaster*.

### About

The About page gives more details on the algorithm and datasets used, provides literature references and answers what we expect to be frequently asked questions, including contact details.

## DISCUSSION

RNAct provides an easy-to-use view of protein–RNA interactions in model organisms. It is intended to grow, both in terms of the number of species covered (currently human, mouse and yeast) and in terms of the experimental datasets provided. We hope our database will be particularly useful for studying gene regulatory events and networks at the post-transcriptional level (30). In addition to protein-centric datasets, recently published interactomes for the MALAT1, NEAT1 and NORAD long non-coding RNAs (lncRNAs) from a mass spectrometry-based method make it likely that additional RNA-centric datasets will be published in the near future (31). We are actively implementing features such as flagging interactions which are experimentally validated at low throughput, and allowing users to add articles supporting a given interaction. Interactions supported by the presence of an RNA-binding domain and its corresponding motifs are also intended to be highlighted in future (32). Additionally, we are considering to report the predicted binding regions for each interaction from *cat*RAPID, similar to a CLIP binding profile, although this would require us to upgrade our server infrastructure due to the terabytes of data involved for all pairwise interactions. In summary, RNAct provides easy access to genome-scale protein–RNA interaction predictions with useful supporting annotation and experimental interaction evidence.

## References

[1] Errichelli L., Dini Modigliani S., Laneve P., Colantoni A., Legnini I., Capauto D., Rosa A., De Santis R., Scarfò R., Peruzzi G. FUS affects circular RNA expression in murine embryonic stem cell-derived motor neurons. Nat.Commun. 2017; 8:14741. 28358055 10.1038/ncomms14741 PMC5379105

[2] Castello A., Fischer B., Hentze M.W., Preiss T. RNA-binding proteins in Mendelian disease. Trends Genet. 2013; 29:318–327. 23415593 10.1016/j.tig.2013.01.004

[3] Gerstberger S., Hafner M., Tuschl T. A census of human RNA-binding proteins. Nat. Rev. Genet. 2014; 15:829–845. 25365966 10.1038/nrg3813 PMC11148870

[4] Hentze M.W., Castello A., Schwarzl T., Preiss T. A brave new world of RNA-binding proteins. Nat. Rev. Mol. Cell Biol. 2018; 19:327–341. 29339797 10.1038/nrm.2017.130

[5] Kramer K., Sachsenberg T., Beckmann B.M., Qamar S., Boon K.-L., Hentze M.W., Kohlbacher O., Urlaub H. Photo-cross-linking and high-resolution mass spectrometry for assignment of RNA-binding sites in RNA-binding proteins. Nat. Methods. 2014; 11:1064–1070. 25173706 10.1038/nmeth.3092 PMC6485471

[6] Conrad T., Albrecht A.-S., de Melo Costa V.R., Sauer S., Meierhofer D., Ørom U.A. Serial interactome capture of the human cell nucleus. Nat. Commun. 2016; 7:11212. 27040163 10.1038/ncomms11212 PMC4822031

[7] Castello A., Fischer B., Frese C.K., Horos R., Alleaume A.-M., Foehr S., Curk T., Krijgsveld J., Hentze M.W. Comprehensive identification of RNA-Binding domains in human cells. Mol. Cell. 2016; 63:696–710. 27453046 10.1016/j.molcel.2016.06.029 PMC5003815

[8] Marchese D., de Groot N.S., Lorenzo Gotor N., Livi C.M., Tartaglia G.G. Advances in the characterization of RNA-binding proteins. Wiley Interdiscip. Rev. RNA. 2016; 7:793–810. 27503141 10.1002/wrna.1378 PMC5113702

[9] Castello A., Hentze M.W., Preiss T. Metabolic enzymes enjoying new partnerships as RNA-Binding proteins. Trends Endocrinol. Metab. 2015; 26:746–757. 26520658 10.1016/j.tem.2015.09.012 PMC4671484

[10] Jain S., Wheeler J.R., Walters R.W., Agrawal A., Barsic A., Parker R. ATPase-Modulated stress granules contain a diverse proteome and substructure. Cell. 2016; 164:487–498. 26777405 10.1016/j.cell.2015.12.038 PMC4733397

[11] Sloan C.A., Chan E.T., Davidson J.M., Malladi V.S., Strattan J.S., Hitz B.C., Gabdank I., Narayanan A.K., Ho M., Lee B.T. ENCODE data at the ENCODE portal. Nucleic Acids Res. 2016; 44:D726–D732. 26527727 10.1093/nar/gkv1160 PMC4702836

[12] Hu B., Yang Y.-C.T., Huang Y., Zhu Y., Lu Z.J. POSTAR: a platform for exploring post-transcriptional regulation coordinated by RNA-binding proteins. Nucleic Acids Res. 2017; 45:D104–D114. 28053162 10.1093/nar/gkw888 PMC5210617

[13] Stoiber M.H., Olson S., May G.E., Duff M.O., Manent J., Obar R., Guruharsha K.G., Bickel P.J., Artavanis-Tsakonas S., Brown J.B. Extensive cross-regulation of post-transcriptional regulatory networks in Drosophila. Genome Res. 2015; 25:1692–1702. 26294687 10.1101/gr.182675.114 PMC4617965

[14] Mittal N., Scherrer T., Gerber A.P., Janga S.C. Interplay between posttranscriptional and posttranslational interactions of RNA-binding proteins. J. Mol. Biol. 2011; 409:466–479. 21501624 10.1016/j.jmb.2011.03.064

[15] Marchese D., Botta-Orfila T., Cirillo D., Rodriguez J.A., Livi C.M., Fernández-Santiago R., Ezquerra M., Martí M.J., Bechara E., Tartaglia G.G. Discovering the 3′ UTR-mediated regulation of alpha-synuclein. Nucleic Acids Res. 2017; 45:12888–12903. 29149290 10.1093/nar/gkx1048 PMC5728410

[16] Cirillo D., Blanco M., Armaos A., Buness A., Avner P., Guttman M., Cerase A., Tartaglia G.G. Quantitative predictions of protein interactions with long noncoding RNAs. Nat. Methods. 2017; 14:5–6. 10.1038/nmeth.4100 28032625

[17] Bellucci M., Agostini F., Masin M., Tartaglia G.G. Predicting protein associations with long noncoding RNAs. Nat. Methods. 2011; 8:444–445. 21623348 10.1038/nmeth.1611

[18] Cirillo D., Agostini F., Klus P., Marchese D., Rodriguez S., Bolognesi B., Tartaglia G.G. Neurodegenerative diseases: quantitative predictions of protein-RNA interactions. RNA. 2013; 19:129–140. 23264567 10.1261/rna.034777.112 PMC3543085

[19] Agostini F., Cirillo D., Bolognesi B., Tartaglia G.G. X-inactivation: quantitative predictions of protein interactions in the Xist network. Nucleic Acids Res. 2013; 41:e31. 23093590 10.1093/nar/gks968 PMC3592426

[20] The UniProt Consortium UniProt: the universal protein knowledgebase. Nucleic Acids Res. 2017; 45:D158–D169. 27899622 10.1093/nar/gkw1099 PMC5210571

[21] Harrow J., Frankish A., Gonzalez J.M., Tapanari E., Diekhans M., Kokocinski F., Aken B.L., Barrell D., Zadissa A., Searle S. GENCODE: the reference human genome annotation for The ENCODE Project. Genome Res. 2012; 22:1760–1774. 22955987 10.1101/gr.135350.111 PMC3431492

[22] Zerbino D.R., Achuthan P., Akanni W., Amode M.R., Barrell D., Bhai J., Billis K., Cummins C., Gall A., Girón C.G. Ensembl 2018. Nucleic Acids Res. 2018; 46:D754–D761. 29155950 10.1093/nar/gkx1098 PMC5753206

[23] Lorenz R., Bernhart S.H., Höner zu Siederdissen C., Tafer H., Flamm C., Stadler P.F., Hofacker I.L. ViennaRNA Package 2.0. Algorithms Mol. Biol. 2011; 6:26. 22115189 10.1186/1748-7188-6-26 PMC3319429

[24] Van Nostrand E.L., Pratt G.A., Shishkin A.A., Gelboin-Burkhart C., Fang M.Y., Sundararaman B., Blue S.M., Nguyen T.B., Surka C., Elkins K. Robust transcriptome-wide discovery of RNA-binding protein binding sites with enhanced CLIP (eCLIP). Nat. Methods. 2016; 13:508–514. 27018577 10.1038/nmeth.3810 PMC4887338

[25] ENCODE Project Consortium An integrated encyclopedia of DNA elements in the human genome. Nature. 2012; 489:57–74. 22955616 10.1038/nature11247 PMC3439153

[26] Brannan K.W., Jin W., Huelga S.C., Banks C.A.S., Gilmore J.M., Florens L., Washburn M.P., Van Nostrand E.L., Pratt G.A., Schwinn M.K. SONAR Discovers RNA-Binding proteins from analysis of Large-scale Protein-Protein interactomes. Mol. Cell. 2016; 64:282–293. 27720645 10.1016/j.molcel.2016.09.003 PMC5074894

[27] Livi C.M., Klus P., Delli Ponti R., Tartaglia G.G. catRAPID signature: identification of ribonucleoproteins and RNA-binding regions. Bioinformatics. 2016; 32:773–775. 26520853 10.1093/bioinformatics/btv629 PMC4795616

[28] Zhang T., Tan P., Wang L., Jin N., Li Y., Zhang L., Yang H., Hu Z., Zhang L., Hu C. RNALocate: a resource for RNA subcellular localizations. Nucleic Acids Res. 2017; 45:D135–D138. 27543076 10.1093/nar/gkw728 PMC5210605

[29] Rodriguez J.M., Rodriguez-Rivas J., Di Domenico T., Vázquez J., Valencia A., Tress M.L. APPRIS 2017: principal isoforms for multiple gene sets. Nucleic Acids Res. 2018; 46:D213–D217. 29069475 10.1093/nar/gkx997 PMC5753224

[30] Dassi E., Re A., Leo S., Tebaldi T., Pasini L., Peroni D., Quattrone A. AURA 2: empowering discovery of post-transcriptional networks. Translation (Austin). 2014; 2:e27738. 26779400 10.4161/trla.27738 PMC4705823

[31] Spiniello M., Knoener R.A., Steinbrink M.I., Yang B., Cesnik A.J., Buxton K.E., Scalf M., Jarrard D.F., Smith L.M. HyPR-MS for multiplexed discovery of MALAT1, NEAT1 and NORAD lncRNA protein interactomes. J. Proteome Res. 2018; 17:3022–3038. 29972301 10.1021/acs.jproteome.8b00189 PMC6425737

[32] Giudice G., Sánchez-Cabo F., Torroja C., Lara-Pezzi E. ATtRACT-a database of RNA-binding proteins and associated motifs. Database. 2016; 2016:baw035. 27055826 10.1093/database/baw035 PMC4823821

